# Case Report: Pathological features of explanted native lungs in a patient with end stage lung disease after resolution of severe COVID19 who underwent successful lung transplantation

**DOI:** 10.3389/fmed.2025.1580570

**Published:** 2025-09-02

**Authors:** Wei Guo, Yan Liu, Minghuan Ge, Xiangchao Ding, Bo Wang, Jian Xu, Xiaoying Chu, Sufang Tian, Huiqing Lin

**Affiliations:** ^1^Department of Pathology, Wuhan University School of Basic Medical Sciences, Wuhan, China; ^2^Department of Pathology, Wuhan University Zhongnan Hospital, Wuhan, China; ^3^Department of Thoracic Surgery, Renmin Hospital of Wuhan Univerity, Wuhan, China; ^4^Department of Urology, Remin Hospital of Wuhan University, Wuhan, China

**Keywords:** COVID-19, diffuse pulmonary fibrosis, lymphocyte infiltration, SARS-COV2, lung transplantation

## Abstract

During the pandemic of COVID-19, lots of features of this disease have been discovered. However, the lung pathology and the correlated clinical features of the patients who recovered from the severe state of COVID-19 are still largely unknown. Especially for those who underwent diffuse alveolar damage (DAD), most of the morphological data were obtained from the autopsy specimens or biopsy samples. In the present report, the pathologic changes in the lungs of a patient who had successfully received lung transplantation at the recovery stage of severe COVID-19 were described. Diffuse alveolar damage, hyperplasia of interstitial fibroblast and alveolar type II epithelial cells, and the filling of macrophages in alveoli were observed. Hyperemia and thickening of blood vessels and interstitial lymphocytic inflammation were also prominent. SARS-COV-2 nuclear capsid was detected spotty in the alveolar epithelial after several times' negative nucleic acid results from his pharyngeal swab specimens. Evidence of combined virus infection, such as Cytomegalovirus, could also be found. A few eosinophils were found in the parenchymal of the lung, which combined with the elevated eosinophils in the blood, might indicate a recovery of this patient. This rare case provides a chance for us to observe the pathological changes in the diffuse fibrosis stage of severe COVID-19, which might help us to further understand how pulmonary fibrosis forms after severe pathogen infection.

## Introduction

The Coronavirus disease 2019 (COVID-19) has caused massive mortality globally. Although plenty of research has been published about the source of the pathogen, the transmission, and the clinical features of this novel infectious disease (1–3), much is still unknown about the pathogenesis, development, and long-term prognosis. Several studies reported the systemic pathological description of severe cases in the early phase (4). However, there was no report about pathological features of advanced lung fibrosis in lived patients to the best of our knowledge. In the present study, we report the clinical and pathological features of a severe case of a patient who recovered from COVID-19 after successful lung transplantation. The duration of the illness was 180 days.

## Clinical course highlights

A 65-year-old male patient who lived in Wuhan city, presented with a high fever and was admitted to the hospital on January 23rd, 2020. His highest temperature was 39.6°C. He had no past medical history of hypertension, chronic obstructive pulmonary disease, or allergic diseases. Ground-glass opacities in his lungs were later detected through computerized tomography (CT). On 16th February, he was admitted to the hospital and was transferred to the intensive care unit (ICU) because of the worsening clinical indicators. On 17th February, he received intubation and mechanical ventilation. From 18th February, venovenous extracorporeal membrane oxygenation (V-V ECMO) was used to provide adjunct support. During his hospitalization, he underwent multiple tests for SARS-CoV-2 nucleic acid using his throat swab specimen, anal swab specimen, and bronchoalveolar lavage, and the results were all negative. However, his serum and swab specimens tested positive for SARS-CoV-2 IgG antibodies. On 6th February, he tested positive for Methicillin-resistant Staphylococcus aureus (MRSA) in his blood culture. Specific IgG for Epstein-Barr (EB) virus and cytomegalovirus (CMV) were also found in his serum specimen. He was given a comprehensive treatment that included antibacterial, anti-fungal, antiviral, and supportive treatment. On 20th March, he was tested as coinfection with carbapenem-resistant *Acinetobacter baumannii* (CRAB). On 20th April, he was evaluated for the clinical indices and received double lung transplantation. After 92 days of rehabilitation, he made a complete recovery by the 21st of July and was discharged from the hospital.

## Radiological and laboratory findings

**Figure 1:**
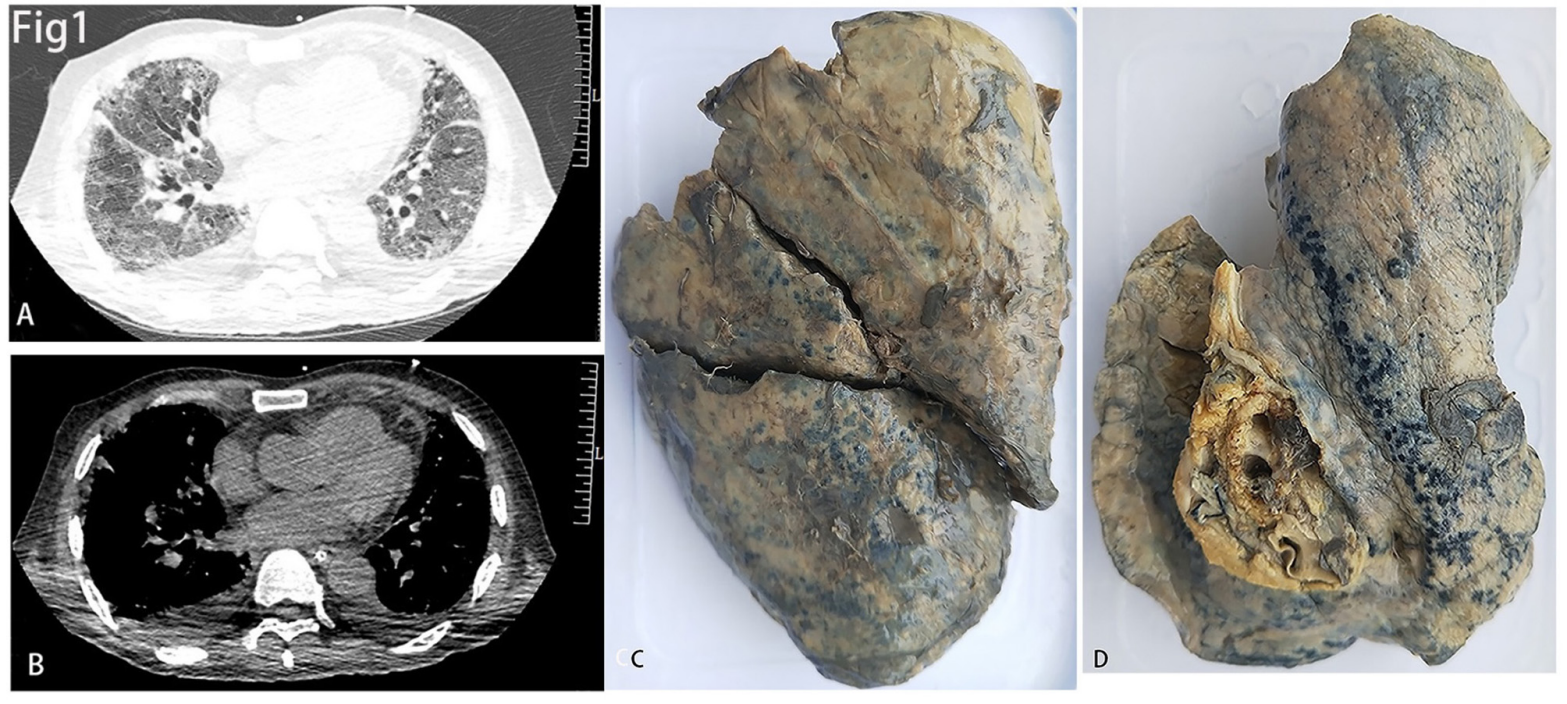
**(A, B)** The lung window **(A)** and mediastinal window **(B)** of CT showed diffused fibrosis of both lungs. **(C, D)** showed decreased size of the right **(C)** and left **(D)** lungs.

CT images of the lungs showed ground-glass opacities, septal thickening, and traction bronchiectasis which indicated diffuse fibrosis in both lungs (Figures 1A, B). The gross inspection showed shrunken lungs with characteristic stiffness and a tinge of yellow color (Figures 1C, D).

The dynamic laboratory results before and after lung transplantation are listed in Table 1. The pathological progression of ARDS in lungs and associated laboratory-pathological characteristics are summarized in Table 2. Before transplantation, his peripheral white blood cells were elevated. Detailed analysis showed an increase in neutrophils and eosinophils, while the lymphocyte count was close to the lower limit of the normal reference in the peripheral blood. Amongst the subsets of lymphocytes, the T lymphocyte value was close to the lower limit of the normal range before transplantation. After transplantation and long-term treatment, the number of neutrophils dropped to the normal range, while the eosinophils were slightly higher than normal. He presented with lymphocytopenia before discharging from the hospital, while the counts of subgroups of T lymphocytes were even lower than those prior to the transplantation. At the same time, the values of C reaction protein (CRP) and hypersensitivity CRP were significantly higher than normal before and after the transplantation. In humoral immunity, only complement C3 had a lower value than the normal range before and after transplantation, while other biochemical parameters showed at normal ranges. The coagulation function was also evaluated. Prothrombin time was slightly longer than the normal reference, while activated partial thromboplastin time (APTT), fibrin, and D-dimer showed higher values than normal.

**Table 1:** Laboratory findings prior and post lung transplantation.

**Date**	**04/17/20**	**04/22/20**	**04/24/20**	**06/16/20**	**07/11/20**	**07/20/20**
White blood cell (3.5–9.5 × 109/L)	12.8	24.58	36.87	20.4	4.9	5.2
Neutrophils (1.8–6.3 × 109/L)	8.52	23.21	34.14	17.08	4.17	3.55
Eosinophils (0.02–0.52 × 109/L)	2.15	0.02	1.37	0.01	0.28	0.87
Lymphocytes (1.1–3.2 × 109/L)	1.16	0.52	0.98	0.44	0.27	0.57
Hypersensitivity CRP (0–3 mg/L)	>5	>5	>5	>5	N/A	N/A
C reaction protein (0–5 mg/L)	49.5	26	68.8	27.9	24.4	37
Prothrombin time (9–13 S)	13.9	15	13.7	13.9	18.4	13.9
APTT (S) (25–31.3s)	78.1	32.9	35.8	37.2	39.8	41.6
FIBRIN (2–4 g/L)	2.98	1.51	1.91	1.85	0.63	1.63
AT III (80–120%)	44.10%	44.40%	40.10%	50.20%	50.20%	48.30%
D-dimer (0–0.55 mg/L)	11.76	5.35	7.22	3.57	8.2	10.91
**Evidence for infection**						
Nucleic acid for SARS-CoV-2	Negative	Negative	Negative	Negative	Negative	Negative
Tissue culture for other pathogens	N/A	N/A	N/A	ABA	Negative	Negative
**Subgroups of lymphocytes**						
CD3(727–2737 count/uL)	806	378	613	283	243	334
CD4 (404–1612 count/uL)	510	256	429	153	129	124
CD8 (220–1129 count/uL)	310	122	196	136	112	212
CD4/CD8 (0.9–2.0)	1.65	2.09	2.19	1.13	1.16	0.58
CD19 (80–616 count/uL)	49	37	77	36	8	12
CD56 (84–724 count/uL)	180	47	69	66	94	88
**Humoral immunity**						
IgG (8–16 g/L)	7.35	7.95	12.8	15.5	7.45	11
IgM (0.4–3.45 g/L)	0.561	0.646	0.849	0.593	0.774	0.895
IgA (0.76–3.9 g/L)	0.975	1.34	1.44	0.803	0.96	0.8
IgE (< 100 IU/mL)	55.6	88.7	132	21.2	32.4	18.8
Complement C3 (0.81–1.6 g/L)	0.667	0.286	0.418	0.454	0.621	0.55
Complement C4 (0.1–0.4 g/L)	0.207	< 0.067	0.091	0.169	0.184	0.163

ABA, *Acinetobacter baumannii*; APTT, activated partial thromboplastin time; CRP, creaction protein.

**Table 2:** Pathological findings.

**Category**	**Pathological features**
Gross features	- Bilateral shrunken lungs with stiffness and yellowish discoloration
Histopathology findings	- Diffuse alveolar damage (DAD) with fibrotic NSIP pattern Fibroblast hyperplasia, and parenchymal remodeling - Hyperemia, intra-alveolar hemorrhage, and mucous plugs - ATII cell hyperplasia and bronchial epithelial metaplasia - Focal atypical alveolar epithelial hyperplasia - Prominent empyema in small bronchia and - pulmonary bullae formation
Vascular changes	- Small vessel wall thickening without vasculitis/thrombosis - Expanded vascular intima (confirmed by elastic staining)
Inflammatory infiltrate	- Lymphocytes (predominantly CD3 + T-cells, CD4+:CD8+ = 1:1–1:2), eosinophils, histiocytes - Perivascular inflammation with lymphoid follicles - CD163+ macrophages in interstitium/alveoli - Scattered plasma cells (CD38+), rare NK/T cells
Cytopathic/viral markers	- CMV+ intranuclear inclusions - SARS-CoV-2 nucleocapsid protein in alveolar/bronchial epithelia
Other findings detected by immunohistochemistry	- Squamous metaplasia (P40+/TTF-1+ co-expression) - Diffuse IL-6, IFN-γ, IL-17 expression - Focal Granzyme B+ cytotoxic cells near airways - Minimal PD-1/PD-L1 expression
Key absent features	- No hyaline membranes or fibrinoid alveolar exudates - No vasculitis/thrombi

## Pathological findings

**Figure 2:**
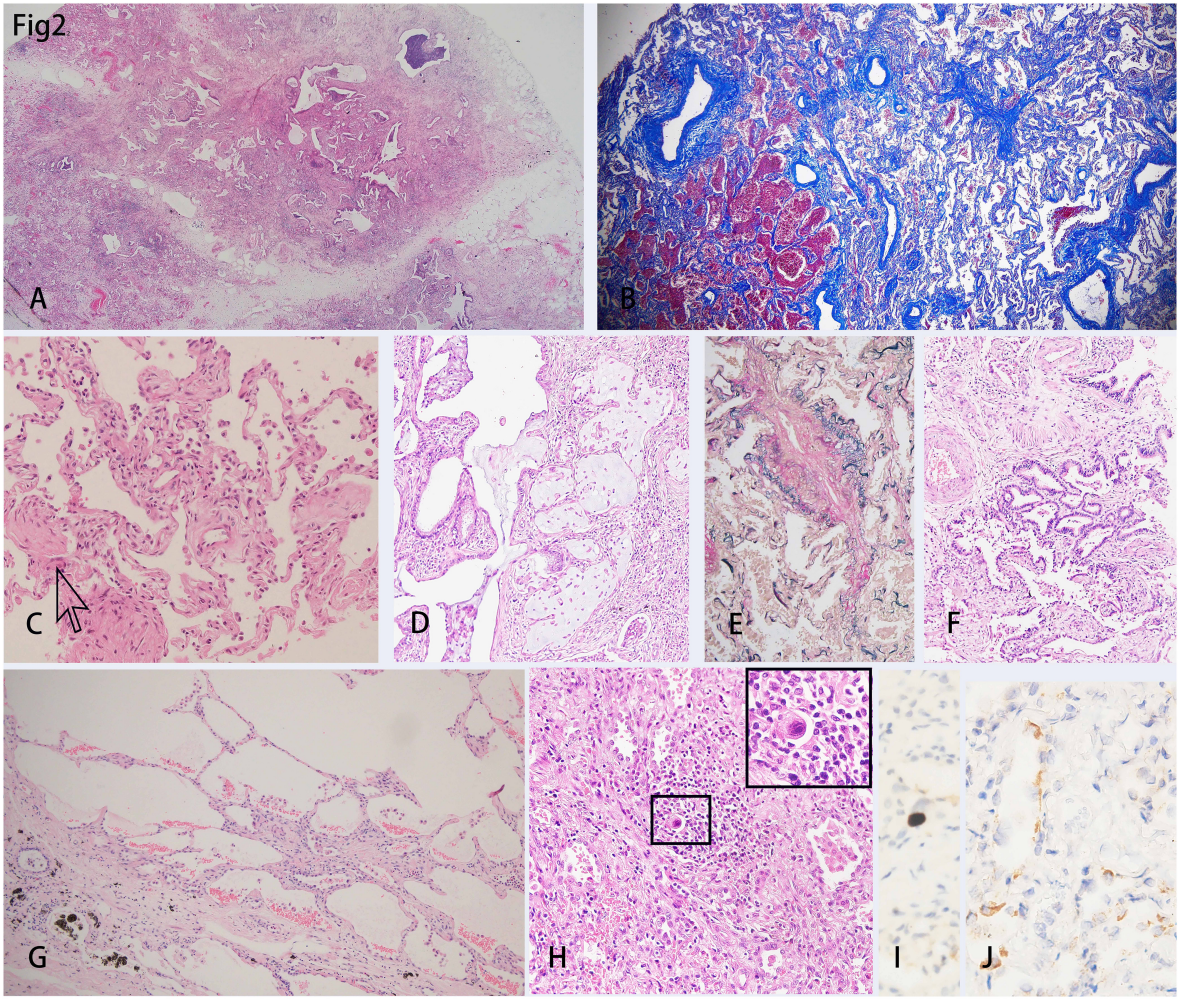
H&E staining of the lungs showed diffused damage and inflammation. **(A)** Collagens extended from the pleura to the parenchyma of the lung, which separated the lung parenchyma into several lobules. **(B)** Masson trichrome staining showed the diffusely distributed fibers in the mesenchyme. **(C)** Intra-alveolar fibroblast nodules could be scarcely found (indicated by the arrow). **(D)** Intra-alveolar mucus plug could be found. **(E)** Elastic fibers staining showed the thickened intima of the blood vessel wall. **(F)** There was a cluster of alveolar epithelia undergoing atypical intra-epithelial hyperplasia (AIH). **(G)** Bullae of the lung was shown under the pleura. **(H)** An intranuclear inclusion body was shown in the background of diffuse inflammation and was amplified in the inserted right upper rectangle. **(I)** Immunohistochemistry showed a cell infected with Cytomegalovirus (CMV). **(J)** The SARS-CoV-2 nucleocapsid protein was detected in the epithelial of a few alveoli by immunohistochemistry staining.

Microscopically, the lungs exhibited diffuse alveolar damage (DAD) and a fibrotic non-specific interstitial pneumonia pattern, i.e., diffuse involvement of the alveolar walls with thickening, fusion, and simplification (5). Dense collagen and fibers were diffusely distributed in the septa of the alveoli (Figures 2A, B). In this case, neither was the fibrinoid exudate in the alveoli nor the hyaline membrane identified, unlike the previously reported cases in the acute phase. Only a few intra-alveolar fibroblast nodules were found (Figure 2C). Some alveolar spaces were filled with mucous plugs (Figure 2D). The walls of small blood vessels were significantly thickened. However, the vasculitis and thrombus were not presented (Figure 2E). Not only were the clusters of alveolar epithelia that underwent atypical hyperplasia seen (Figure 2F), but pulmonary bulla at the margin of the lungs was also observed (Figure 2G). Various amounts of lymphocytes, eosinophils, and histiocytes infiltrated the interstitial tissue. Some of the remaining alveoli were filled with macrophages along with the desquamated alveolar epithelium. A few intranuclear inclusions were identified and proven by the immune-histochemistry staining for cytomegalovirus (CMV) (Figures 2H, I). The SARS-CoV-2 nucleocapsid protein was detected positive in the epithelial cells of a few alveoli and bronchia (Figure 2J).

**Figure 3:**
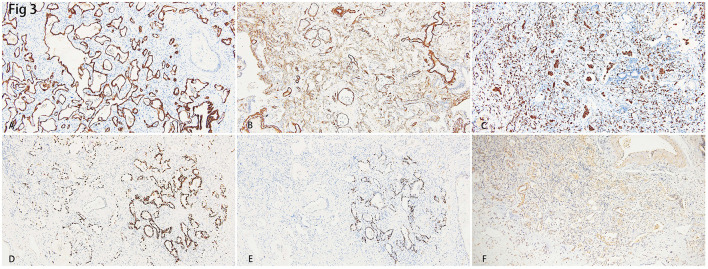
**(A)** Immunohistochemistry staining of cytokeratin (CK) depicted the epithelial of alveoli and bronchus. **(B)** Smooth Muscle Actin (SMA) showed hyperplasia of interstitial fibroblasts. **(C)** Intra-alveoli and interstitial distribution of CD163 positive macrophages. **(D)** Thyroid transcription factor-1 (TTF-1) showed hyperplasia of alveolar epithelial II (AT-II). **(E)** The staining of the squamous epithelial marker, P40, showed a cluster of alveoli that underwent squamous metaplasia. **(F)** Diffuse expression of IL-6 indicated a heavy inflammation.

Immune-histochemistry staining showed that SMA was expressed in the interstitium of the lungs (Figures 3A, B), which indicated the hyperplasia of fibroblasts. CD163+ macrophages were distributed in both the interstitium and in some alveolar spaces (Figure 3C). The co-expression of P40, which is the marker of squamous epithelia, and ATII epithelial marker TTF-1, proved the metaplasia of squamous epithelium in several alveoli (Figures 3D, E). Inflammatory cytokines such as IL-6 (Figure 3F), IFN- γ, and IL-17 (data not shown) showed diffused distribution in the parenchyma of the lungs.

**Figure 4:**
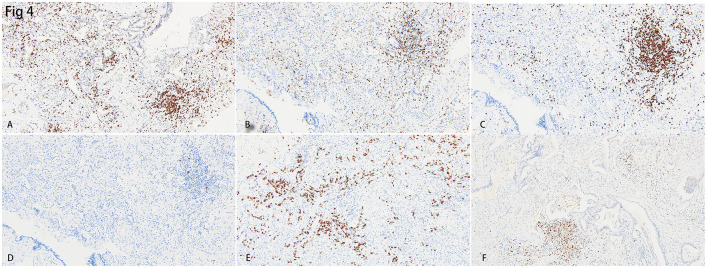
Immunohistochemistry staining for the inflammatory cells in the lungs. **(A)** Diffusely distributed CD3+ T lymphocytes were identified. B&C. CD4+ T lymphocytes **(B)** were lesser than CD8+ T lymphocytes **(C)** in the interstitium of the lung. **(D)** CD20+ B lymphocytes were hardly found in the lung. **(E)** CD38+ plasma cells were scattered in the interstitium of the lung. **(F)** Cytotoxic cytokine, granzyme B, was identified in the region near small bronchia.

Additional immunohistochemistry staining was performed to further delineate the infiltrated inflammatory cells. Most of the lymphocytes that infiltrated the septa of alveoli were CD3+ T lymphocytes (Figure 4A), and a few CD20+ B lymphocytes were scattered in the lymphoid follicles (Figure 4D). The ratio of CD4+ T lymphocytes to CD8+ T lymphocytes was between 1:1 and 1:2 (Figures 4B, C). There were also CD38+ plasma cells distributed along with parts of the interstitium of the alveoli (Figure 4E). Cytotoxic molecules, such as Granzyme B were majorly distributed adjacent to the bronchia and alveoli (Figure 4F).

## Discussion

Pulmonary pathology of SARS-CoV-2 infection encompasses congestion in small blood vessels, hyaline degeneration, fibrinous exudation in the alveoli, as well as the infiltration of T lymphocytes, neutrophils, and eosinophils in the interstitium (6). Most of these reports were from the autopsies conducted on the patients who died in the acute phase. Although a transplanted case in severe COVID-19 was reported, the major morphology of that case also showed diffuse alveolar damage and early fibrosis but not late-stage fibrosis (7). Becker et al. (8) conducted transcriptomic revealed that extensive alveolar damage in severe COVID-19 cases is closely associated with vascular leakage, which plays a crucial role in the progression of pulmonary fibrosis. To check whether mild cases of COVID-19 develop into lung fibrosis in the long run still requires a long-term follow-up and large-scale investigation.

Histologically, the patterns of fibrosis in the present COVID-19 case were similar to idiopathic pulmonary fibrosis (IPF), i.e., diffused fibrosis, interstitial inflammation, thickening of the blood vessel walls, and hyperplasia of AT-II cells. However, IPF is a chronic disease that spread over a period of decades (9). In the present case, the formation of diffuse lung fibrosis came up in only several months, which progressed significantly faster than IPF. The interstitial inflammation and diffused fibrosis were prominent, while there were no findings of typical honeycomb fibrosis in the present case. These findings could be evidence of late-stage DAD caused by the virus (4, 10). COVID-19-induced fibrotic tissue remodeling occurs rapidly and demonstrates at least partial reversibility in most survivors. Research indicates that pro-fibrotic CD163+ macrophages are significantly induced and rapidly accumulate following SARS-CoV-2 exposure, directly contributing to pulmonary fibrosis. This finding is supported by autopsy reports of severe COVID-19-associated acute respiratory distress syndrome (ARDS), which reveal extensive deposition of types 1, 3, and 4 interstitial collagens in the lungs (11, 12). The regulation of inflammation and correlated responses of lung repair in different injury backgrounds caused different patterns of lung fibrosis. In this case, the inflammation and necrosis caused by the co-infection of bacteria could not be ignored because bacterial sepsis might not only destroy the structure of alveolar walls but also cause fibrotic repair (13, 14). Bacterial infection significantly promotes the progression of pulmonary fibrosis through the activation of multiple critical signaling pathways, such as the TGF-β/Smad signaling pathway (15), and TLR4/NF-κB inflammatory pathway (16). Furthermore, the application of mechanical ventilation, ECMO, and the usage of some medications such as corticosteroids and immune regulators should also be considered as the factors that accelerate lung fibrosis (17).

The inflammatory infiltration throughout the lung was prominent, corresponded to his elevated peripheral white blood cell profile, and indicated a general mobilization of the immune system to cope with the virus and promote lung repair and fibrosis. Although there were reports about neutrophils causing lung fibrosis (18), however, we didn't find prominent neutrophil infiltration presented in his lungs. The infiltrated inflammatory cells were majorly lymphocytes, eosinophils, and monocyte-macrophages. There was a formation of a few lymph follicles which consisted of dominantly CD3+ T cells and a small portion of CD20+ B lymphocytes. In the present case, we found that although the ratio of CD4/CD8 T lymphocytes was lower than 1:1, it was close to the ratio in the blood. This seemed to indicate a recovery of the immune system.

It was reported that the DAD caused by SARS-CoV-2, presented as a proliferation of blood vessels, vascular congestion, and thickening of the blood vessel walls. In the autopsy reports of COVID-19 patients, diffuse thrombi and endothelialitis were detected in the lungs (19). However, in this case, the thrombi and endothelialitis were not discovered, and this is corresponding to the changing process of his prothrombin time. Instead, diffused thickened blood vessel walls were prominent, which was according to the previous reports (20). These changes indicated that the injured blood vessels underwent repair after the virus clearance, which in turn might cause pulmonary hypertension and may contribute to the endothelial transition to mesenchymal in the long run. Both thickened blood vessels and pulmonary hypertension could also be found in IPF. Endothelial colony-forming cells (ECFCs) have been identified as an elevated liquid biomarker in patients with idiopathic pulmonary fibrosis (IPF) (21). These cells promote both angiogenesis and fibroblast proliferation through the SDF-1/CXCR4 signaling pathway, thereby contributing to the pathological progression of IPF (22). Furthermore, the proliferation of endothelial colony-forming cells (ECFCs) has been closely linked to angioproliferative mechanisms in pulmonary arterial hypertension (PAH). This severe cardiopulmonary disorder is pathologically characterized by progressive remodeling of pulmonary vasculature and the development of precapillary pulmonary hypertension (21).

SARS-COV-2 causes DAD in severe cases of COVID-19, which is lethal to most patients.

We cannot conclude that this patient showed the typical lung pathology of COVID-19, but the features of this case, together with other reports will help us to understand the clearer nature of lung fibrosis after severe infection of SARS-COV-2.

## Materials and methods

Fresh samples of both lungs of the patient were inspected immediately after resection by Wuhan Institute of Virology, Chinese Academy of Sciences. The outcome of the test was negative for the SARS-CoV-2 live virus and nucleic acid (data not shown). For pathologic inspection, each lung lobe was sampled for 4–6 blocks after fixing with 10% neutral formalin for at least 72 h. Routine hematoxylin-eosin staining and immunohistochemistry were performed. The antibodies against CD3, CD4, CD8, CD20, CD56, CD68, CD163, TIA-1, Granzyme B, TTF-1, CK, and P40 were purchased from Zhongshan golden bridge Biotechnology, Beijing. Antibodies against PD-1, PD-L1 (Dako 22c3), Cytomegalovirus, and EBER RNA CISH Probe were purchased from Agilent Technologies, Inc. Antibody against SARS-CoV-2 nucleocapsid protein was purchased from Abcam Public Limited Company, MA, USA. Immunohistochemistry was performed with the Bond-max automated Immunohistochemistry instrument (Leika Biosystems, U.S.) following the manufacturer's instructions.

## Data Availability

The original contributions presented in the study are included in the article/Supplementary material, further inquiries can be directed to the corresponding authors.

## Supplementary material

Supplementary Figure 1 The clinical flow of the COVID-19 patient.
