# Supplementary figures and images for: Case Report: Pathological features of explanted native lungs in a patient with end stage lung disease after resolution of severe COVID19 who underwent successful lung transplantation
Source: Front Med (Lausanne). 2025 Sep 2;12:1580570. doi: 10.3389/fmed.2025.1580570 (PMC12436437; doi:10.3389/fmed.2025.1580570)

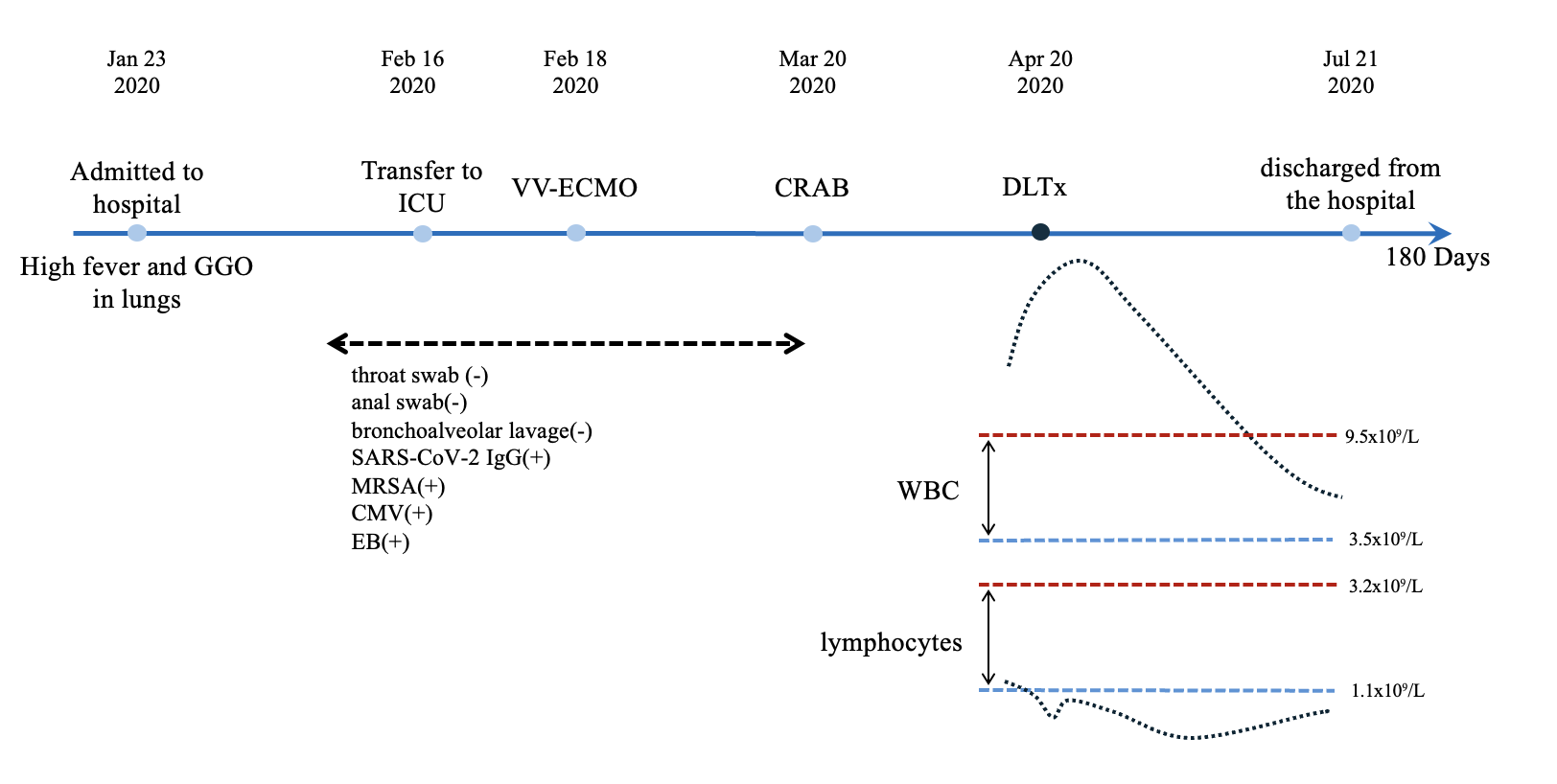

Supplement: Supplementary Figure 1 — The clinical flow of the COVID-19 patient. [file Image_1.jpeg]
